# Cervical anaerobic vertebral osteomyelitis following surgical tracheotomy: a case report

**DOI:** 10.1186/s12879-019-4291-x

**Published:** 2019-07-22

**Authors:** Romaric Larcher, Camille Maury, Jonathan Charbit, Helene Jean-Pierre, Vincent Le Moing, Kada Klouche, Xavier Capdevila

**Affiliations:** 1 Intensive Care Medicine Department, Lapeyronie Hospital, Montpellier, France; 2 Intensive Care Unit, Anaesthesiology and Intensive Care Department, Lapeyronie Hospital, Montpellier University Hospital, Montpellier, France; 3 Bacteriology Department, Arnaud de Villeneuve Hospital, Montpellier University Hospital, Montpellier, France; 4 Tropical and Infectious Diseases Department, Saint Eloi Hospital, Montpellier University Hospital, Montpellier, France; 5 INM, University Montpellier, INSERM, Montpellier, France

**Keywords:** Anaerobic, Anaerobes, Cervical vertebral osteomyelitis, Tracheotomy, Tracheostomy, Trauma patient, case report

## Abstract

**Background:**

We report a rare case of anaerobic vertebral osteomyelitis associated with surgical tracheotomy which has never been reported to the best of our knowledge.

**Case presentation:**

A healthy 39-year-old man was admitted to intensive care for a severe brain trauma injury where a surgical tracheotomy was performed. He was discharged to a rehabilitation centre after 54 days hospital stay. During rehabilitation, he developed progressive and febrile tetraplegia associated with cervical pain, requiring an intensive care readmission. A polymicrobial anaerobic bloodstream infection was revealed and magnetic resonance imaging diagnosed cervical vertebral osteomyelitis. Both the type of anaerobic micro-organisms found and the timing of the symptoms strongly suggest that the surgical tracheotomy was responsible for this rare case of cervical vertebral osteomyelitis. The patient was successfully treated by a prolonged antimicrobial therapy and by surgical laminectomy.

**Conclusions:**

Tracheotomy may generate anaerobic bacteraemia and related osteomyelitis in the specific setting of severe trauma patients. Clinicians should consider anaerobic vertebral osteomyelitis when they are confronted with a febrile tetraplegia after tracheotomy.

## Background

Bacteraemia following tracheotomy occurs in approximately 20% of procedures, but septic metastasis remains rare [1]. Despite the advent of molecular identification and matrix-assisted laser desorption ionization time-of-flight (MALDI-TOF) mass spectrometry, anaerobic bacteraemia, frequently polymicrobial, and related bone and joint infections are yet underestimated [2]. We report herein a case of anaerobic cervical vertebral osteomyelitis as a delayed complication of surgical tracheotomy.

## Case presentation

A healthy 39-year-old man was admitted to the intensive care unit (ICU) for a severe trauma brain injury and a moderate thoracic trauma caused by a motorcycle accident. After initial computed tomography (CT-scan), the patient had immediate craniectomy and hematoma evacuation surgery with an antibiotic prophylaxis by cefazolin. An *Enterobacter aerogenes* ventilator associated pneumonia was diagnosed on day 14, and treated by cefepim for 8 days. After 30 days in ICU, neurological examination showed a minimally conscious state and right lower limb monoplegia. A percutaneous endoscopic gastrostomy (PEG) was therefore positioned on day 31 with an antibiotic prophylaxis by amoxicillin/clavulanate (2 g). A surgical tracheotomy was also performed on day 33, as we use to do in our trauma ICU. Antibiotic prophylaxis was not administered during this procedure. There was no local complication or fever immediately around the surgical tracheostomy. The patient was weaned on day 40. On ICU discharge (day 54), neurological examination was unchanged. The patient was thereafter transferred to a rehabilitation centre.

**Fig. 1:**
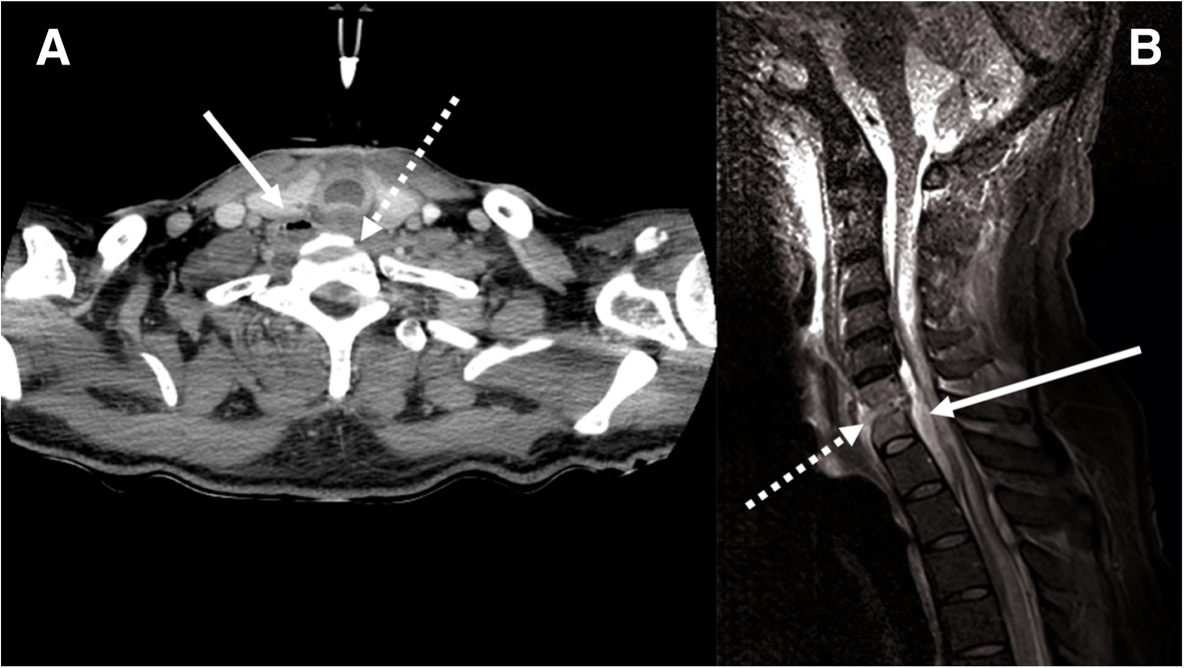
**a** Cervical computed tomography scanner: erosion of C6 vertebral body endplate (white dotted arrow) associated with epidural and right paravertebral hydro-aeric collections (white arrow). **b** Cervical and medullar magnetic resonance imaging: *STIR* hypersignal of disc with irregularity and erosion of C6-C7 vertebral body endplates (white dotted arrow), and epidural collections (white arrow)

During the second week of rehabilitation stay, fever, purulent pulmonary secretions, dyspnoea and cervical pain with progressive tetraplegia appeared. The patient was rapidly readmitted to the ICU where a broad-spectrum antimicrobial therapy including piperacillin/tazobactam (4.5 g four times a day), amikacin (30 mg/kg) and linezolid (600 mg twice a day) was initiated after multiple bacteriological samples. CT-scan and Magnetic Resonance Imaging (MRI) revealed C6-C7 vertebral osteomyelitis associated with a C2-T3 epidural abscess, a right C6-C7 paravertebral abscess and a cervical myelitis (Fig. 1). Among bacteriologic assessment, *Parvimonas micra, Dialister pneumocintes* and *Veillonella parvela* were isolated in three blood cultures. The anaerobes were susceptible to amoxicillin/clavulanate, imipenem and metronidazole, but resistant to clindamycin. The antimicrobial therapy was thus targeted to metronidazole (500 mg every 8 h) and amoxicillin/clavulanate (2 g every 4 h). Cultures of arterial and central venous catheters were negative. Complementary investigations by oral examination, nasofibroscopy, facial and cervical CT-scan, and transoesophageal echocardiography did not reveal any infection source. Two weeks later, cervical vertebrae laminectomy was performed because of persistent fever and tetraplegia. Surgical vertebral biopsies cultures performed under antimicrobial therapy were negative. After laminectomy and a total of 5 weeks of antimicrobial therapy, the patient was apyretic and recovered mobility of the four limbs. He was discharged to the rehabilitation centre after 44 days, treated by amoxicillin/clavulanate and rifampin (900 mg a day) according to susceptibility test. Treatment was well tolerated.

## Discussion and conclusions

Bacteraemia following tracheotomy may occur in about 8–9% of case [1]. However septic metastasis remains uncommon. We report a case of cervical anaerobic vertebral osteomyelitis which was probably secondary to a surgical tracheotomy procedure. Such a deleterious effect of tracheotomy has never been reported at the best of our knowledge.

Vertebral osteomyelitis whore diagnosis was based on fever, tetraplegia, elevated CRP, and confirmed by bloodstream infection contemporaneous of typical MRI, according to international Guidelines [3]. The fact that surgical vertebral biopsies were negative did not rule out this diagnosis since broad spectrum antimicrobial treatment was already administered.

Our main concern in this case was the infection source. Micro-organisms found and timing of clinical findings strongly suggest that the most likely underlying cause of this vertebral osteomyelitis was the surgical tracheotomy. Namely, the presence in blood cultures of *D. pneumosintes,* which is a commensal organism exclusively found in the oral cavity, points towards an oro-pharyngeal source [4]. In addition, sepsis and neurologic symptoms occurred 30 days after this procedure, which is consistent with usual timing of vertebral osteomyelitis, a diagnosis often delayed by several months [5]. Alternative sources of infection appear less likely. Indeed, craniectomy was performed with cefazolin prophylaxis, more than 60 days before bacteraemia, and no local sign of infection was found on CT-scan or around the surgical site. PEG was performed without complication under amoxicillin/clavulanate prophylaxis. A haematogenous spread mechanism seems more probable than a local septic extension owning that: no direct inoculation or contamination from adjacent soft tissue was evidenced on various investigations by appropriate specialists.

No morbid condition was recorded in the patients’ medical history and glycaemic control was optimized under insulin therapy, but tissue injury following trauma may result in immunoparesis leading to an increased risk of infection [6].

In conclusion, we report a case of cervical vertebral osteomyelitis which appears to be an exceptional complication of surgical tracheotomy. Our observation remains uncommon but may inform clinicians to consider it when progressive febrile tetraplegia after a tracheotomy occurs.

## Data Availability

Data sharing is not applicable to this article as no datasets were generated or analysed during the current study.

## Abbreviations

CT-scan: Computed tomography scanner
ICU: Intensive care unit
MALDI-TOF: Molecular identification and matrix-assisted laser desorption ionization time-of-flight
MRI: Magnetic resonance imaging
PEG: Percutaneous endoscopic gastrostomy
